# Immunomodulatory Effects of the Mycosporine-Like Amino Acids Shinorine and Porphyra-334

**DOI:** 10.3390/md14060119

**Published:** 2016-06-21

**Authors:** Kathrin Becker, Anja Hartmann, Markus Ganzera, Dietmar Fuchs, Johanna M. Gostner

**Affiliations:** 1 Division of Biological Chemistry, Biocenter, Medical University of Innsbruck, Innsbruck 6020, Austria; kathrin.becker@i-med.ac.at (K.B.); dietmar.fuchs@i-med.ac.at (D.F.); 2 Institute of Pharmacy, Pharmacognosy, University of Innsbruck, Innsbruck 6020, Austria; anja.hartmann@uibk.ac.at (A.H.); markus.ganzera@uibk.ac.at (M.G.); 3 Division of Medical Biochemistry, Biocenter, Medical University of Innsbruck, Innsbruck 6020, Austria

**Keywords:** mycosporine-like amino acids, shinorine, porphyra-334, nuclear factor kappa B, indoleamine 2,3-dioxygenase

## Abstract

Mycosporine-like amino acids (MAAs) are secondary metabolites, produced by a large variety of microorganisms including algae, cyanobacteria, lichen and fungi. MAAs act as UV-absorbers and photo-protectants. MAAs are suggested to exert pharmaceutical relevant bioactivities in the human system. We particularly focused on their effect on defence and regulatory pathways that are active in inflamed environments. The MAAs shinorine and porphyra-334 were isolated and purified from the red algae *Porphyra* sp. using chromatographic methods. The effect of MAAs on central signaling cascades, such as transcription factor nuclear factor kappa b (NF-κB) activation, as well as tryptophan metabolism, was investigated in human myelomonocytic THP-1 and THP-1-Blue cells. Cells were exposed to the MAAs in the presence or absence of lipopolysaccharide (LPS). NF-κB activity and the activity of tryptophan degrading enzyme indoleamine 2,3-dioxygenase (IDO-1) were used as readout. Compounds were tested in the concentration range from 12.5 to 200 µg/mL. Both MAAs were able to induce NF-κB activity in unstimulated THP-1-Blue cells, whereby the increase was dose-dependent and more pronounced with shinorine treatment. While shinorine also slightly superinduced NF-κB in LPS-stimulated cells, porphyra-334 reduced NF-κB activity in this inflammatory background. Modulation of tryptophan metabolism was moderate, suppressive in stimulated cells with the lower treatment concentration of both MAAs and with the unstimulated cells upon porphyra-334 treatment. Inflammatory pathways are affected by MAAs, but despite the structural similarity, diverse effects were observed.

## 1. Introduction

The adaptation strategies of organisms that are unavoidably exposed to elevated levels of solar radiation include the production of a broad spectrum of secondary metabolites and pigments as well as the upregulation of stress response and other cytoprotective pathways. An important group of secondary metabolites that are upregulated in response to UV light is mycosporine-like amino acids (MAAs).

MAAs are produced by a large variety of microorganisms including algae, cyanobacteria, lichen and fungi. They gained attention in ecology and pharmacology due to their pronounced UV-absorbing and photo-protective potential as indicated e.g., by high molar extinction coefficients [1,2]. MAAs act as sunscreen by absorbing short wavelength radiation and were shown to counteract DNA impairment and reactive oxygen species (ROS) production, and to protect photosynthetic and several other physiological processes [2,3,4]. The attenuation of photodimer formation is one of the main properties of MAAs. Misonou *et al.* showed that MAA-containing metabolite mixtures of *Porphyra yezoensis* could reduce UV irradiation-induced thymine dimers production [5], and suggested that direct molecule-to-molecule energy transfer processes underlay this protective mechanism.

MAAs are small, water-soluble compounds with a cyclohexenone or cyclohexenimine scaffold, which is usually conjugated to one or two amino acids or amino alcohols in positions C1 and C3 of the basic structure [1]. To date, more than 35 MAAs are known, and, just recently, Hartmann *et al.* could establish a general isolation approach for MAAs, resulting in known derivatives like porphyra-334, shinorine, and palythine, but also a previously unknown MAA, catenelline, whose structure was determined by nuclear magnetic resonance spectroscopy (NMR) [6].

Cytoprotection mediated by phytochemicals is frequently attributed to their antioxidant properties [7], whereby this activity can be exerted either directly or in an indirect manner. In addition, UV-protecting secondary metabolites are often characterized by low toxicity [8]. These promising bioactivities brought MAAs into the research focus of the pharmaceutic industry. Recently, Fernandes *et al.* proposed MAAs as new biocompatible and environmentally friendly materials against UV radiation to challenge the stability and function of cellular structures. Besides low toxicity, photo- and thermo-resistance, MAAs were shown to efficiently protect against UV-A and UV-B radiation [9].

Solar radiation activates numerous biological processes in organisms, whereby the enhanced level of oxidative stress is the major trigger. Rising cellular ROS levels induce redox-sensible signaling cascades in the course of a hierarchic stress response [10]. When oxidative stress is rising to higher levels, activation of transcription factor nuclear factor-κB (NF-κB) and activator protein (AP)-1-signaling dependent cascades, as well as mitogen activated protein kinase (MAPK) signaling, regulate the expression of pro-inflammatory cytokines, chemokines and adhesion molecules [11]. NF-κB is an evolutionary conserved DNA-binding factor, which has a variety of functions in many biological processes, but is of crucial importance in orchestrating initiation, execution and resolution of immune response [12]. Besides its important role in maintaining homeostasis in immune cells, NF-κB is also involved in the expression of prosurvival genes [13]. NF-κB signaling is strongly induced under oxidative stress and is thus one of the most interesting target pathways when searching for anti-inflammatory compounds [14].

Besides NF-κB activation, several other biochemical pathways are induced during the T helper (Th) type 1 immune response. The major Th1-type cytokine interferon-γ (IFN-γ) is the strongest inducer of inflammation-mediated tryptophan catabolism via indoleamine 2,3-dioxygenase (IDO-1), and of neopterin formation via guanosine-5'-triphosphate (GTP)-cylcohydrolase as well as of many other immunobiochemical pathways [15]. IDO-1 is the rate-limiting enzyme in the breakdown of the essential amino acid tryptophan into kynurenine, which represents an anti-proliferative strategy by reducing the growth of invading pathogens and malignant cells. IDO-1 activity can be estimated by the calculation of kynurenine to tryptophan ratio (Kyn/Trp) [16]. In addition, tryptophan depletion and the presence of bioactive kynurenine downstream metabolites regulate T cell responses. Thus, tryptophan metabolism is strongly involved in immunoregulation and induction of tolerance [17].

Both NF-κB signaling and IDO-1 mediated tryptophan breakdown were shown to be affected by a variety of synthetic and natural antioxidants in a suppressive manner [18,19]. The aim of this study was to assess the influence of shinorine and porphyra-334 on the central immunoregulatory pathways of NF-κB activation and tryptophan metabolism in the human myelomonocytic cell line THP-1 and the descending NF-κB/AP-1 reporter cell line THP-1-blue.

## 2. Results

### 2.1. Activation Degree of the Central Inflammation Marker NF-κB

**Figure 1:**
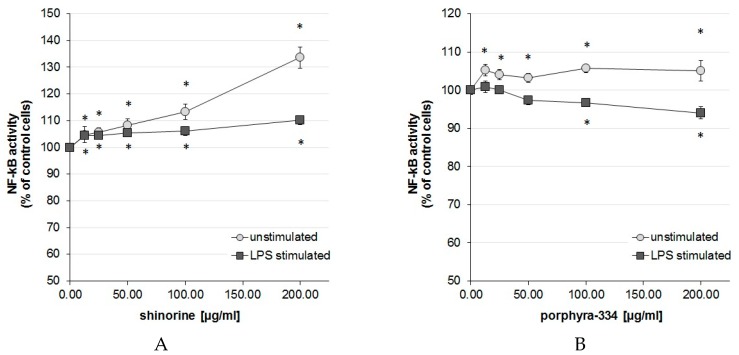
Effect of 48 h incubation with increasing concentrations of shinorine (**A**) and porphyra-334 (**B**) on nuclear factor kappa B (NF-κB) activation in THP-1-Blue cells as indicated by secreted embryonic alkaline phosphatase (SEAP) activity. The enzyme activity measurement is based on the conversion of Quanti-Blue dye, which is added to the cell supernatants. Cells were left either unstimulated (empty spheres) or were stimulated with 1 µg/mL lipopolysaccharide (LPS) (filled squares) after preincubation with mycosporine-like amino acids (MAAs). Results are expressed as mean ± SEM of three independent experiments (* *p* < 0.05 compared to control cells treated with LPS or not).

After 24 h incubation of THP-1-Blue cells with 1 µg/mL lipopolysaccharide (LPS), the activation of NF-κB according to secreted embryonic alkaline phosphatase (SEAP) activity increased significantly up to 5.03 ± 1.90 (mean ± SEM)-fold compared to unstimulated control cells. For easier comparison of the MAA treatment effects, the NF-κB activity of the respective unstimulated and LPS-stimulated control cells is shown as 100% in Figure 1A,B.

Compared to control cells, treatment with shinorine in unstimulated cells activated NF-κB up to 33.63% at the highest treatment concentration (200 µg/mL). In addition, lower concentrations 12.5–100 µg/mL showed a dose-dependent and significant increase in NF-κB activity, starting from 4.78% upwards (*p* < 0.05). Treatment with shinorine in LPS-stimulated cells resulted in a slight but significant superinduction of NF-κB activity compared to LPS treatment alone, e.g., 100 and 200 µg/mL MAA treatment resulted in 6.01% and 10.16% activation compared to LPS-stimulated control cells (Figure 1A).

Treatment with porphyra-334 resulted in a divergent effect depending on the activation level. In unstimulated THP-1-Blue cells, porphyra-334 increased NF-κB activity slightly but significantly at all treatment concentrations (12.5–200 µg/mL) compared to untreated cells; however, the increase was only about 5% and did not follow a dose-dependency. In LPS-stimulated cells, higher concentrations of porphyra-334 reduced NF-κB activity at the two highest concentrations of 100 and 200 µg/mL for 3.25% and 5.94%, respectively. Lower concentrations showed a trend to lower NF-κB activation levels, however without statistical significance (Figure 1B).

### 2.2. Expression of NFKB1 and IL1B

The transcription of the genes nuclear factor kappa B subunit 1 (NFKB1) [20] and interleukin 1B (IL1B) [21] is known to be regulated by NF-κB. qPCR data confirmed higher expression levels of both NF-κB target genes after treatment of THP-1 cells with LPS for 48 h (Table 1A), as indicated by a 3.3-fold induction of NFKB1 and 92.9-fold induction of IL1B expression.

Exposure of THP-1 cells to 200 µg/mL porphyra-334 or shinorine without additional LPS-stimulation did not significantly change the expression of the target genes, although there was a trend towards higher levels of IL1B. Porphyra-334 treatment slightly increased NFKB1 expression (Table 1B). Upon addition of LPS after preincubation of cells with the MAAs, there was no significant change in target gene expression with both compounds (Table 1C).

### 2.3. Effects of Shinorine and Porphyra-334 on Tryptophan Metabolism

After 48 h incubation, the average concentration of tryptophan and kynurenine in supernatants of unstimulated THP-1 was 14.8 ± 0.5 μmol/L (mean ± SEM) and 0.8 ± 0.1 μmol/L, respectively, which results in a Kyn/Trp ratio of 54.5 ± 8.1 μmol/mmol. Upon stimulation of cells with LPS, tryptophan levels decreased, and, in parallel, the kynurenine concentration increased, leading to a Kyn/Trp ratio of 80.7 ± 7.1 μmol/mmol.

**Figure 2:**
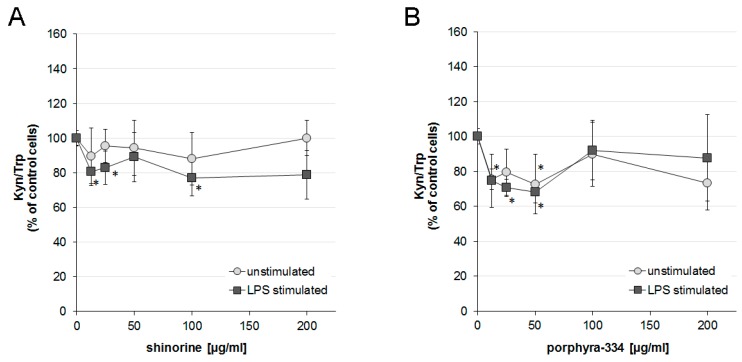
Kynurenine to tryptophan ratio (Kyn/Trp) as a measure of indoleamine 2,3-dioxygenase (IDO-1) activity in the supernatants of unstimulated (empty spheres) or lipopolysaccharide (LPS) (filled squares)-stimulated THP-1-Blue cells after treatment with shinorine (**A**) and porphyra-334 (**B**). Results shown are the mean values ± SEM of three independent experiments run in duplicates (* *p* < 0.05 compared to control cells treated or not with lipopolysaccharide (LPS)).

Treatment of LPS-stimulated cells with both MAAs at lower concentrations decreased Kyn/Trp levels slightly, but significantly. Shinorine decreased the Kyn/Trp at 12.5, 25 and 100 µg/mL treatment compared to LPS-stimulated control samples, with a maximum of 23.1% suppression. Shinorine treatment did not affect unstimulated cells (Figure 2A).

Upon treatment of LPS-stimulated cells with porphyra-334 at concentrations of 25 and 50 µg/mL, Kyn/Trp levels decreased significantly to a maximum of about a 20% decrease for shinorine treatment and of about a 30% decrease for porphyra treatment, in comparison to LPS treated controls. In addition, in unstimulated cells treated with porphyra-334, concentrations of 12.5 and 50 µg/mL lead to a downregulation of Kyn/Trp to a maximum of 72.6%, compared to control cells (100%). The other higher concentrations did not induce significant effects (Figure 2B).

## 3. Discussion

The aim of this study was to investigate the immunomodulatory effects of MAAs in the human myelomonocytic cell line THP-1 and their descendent reporter line THP-1-Blue by focusing on NF-κB activation and tryptophan metabolism, two pathways of central importance in the regulation of inflammatory responses. MAAs have outstanding bioactivities in the organisms, in which they are produced, such as the protection against UV-A/B radiation. Ryu and colleagues showed that porphyra-334 dose-dependently decreased intracellular UV-A-induced ROS generation in human skin fibroblasts [22]. Furthermore, they showed that a treatment with porphyra-334 resulted in decreased levels of matrix metalloproteinases, which were induced by ROS upon UV-A irradiation. Thus, these compounds can influence signaling pathways in the human system, and their effect on prominent pathways in inflammation could give a potential rationale for a pharmaceutic use of MAAs. The promising anti-inflammatory as well as anti-oxidant activities of porphyra-334 and other MAAs tested by de la Coba *et al.* raise interest in the usage of MAAs as therapeutics to protect against UV radiation [23]. The photo-protective capacity of a topical formulation containing porphyra-334 and shinorine isolated from the red alga *Porphyra rosengurttii* was assessed in a mouse study. Maintenance of the antioxidant defence in the skin and expression of 70 kilodalton heat shock protein (Hsp70) were suggested as underlying mechanisms [24]. Schmid *et al.* investigated the UV-A protection activity of MAAs in a human study. A cream containing an extract of the red alga *Porphyra umbilicalis* with an estimated concentration of about 1.4% MAAs (shinorine + porphyra) of the dry weight mass was applied. Besides mediating protection against UV-A radiation, they reported an increase of skin smoothness and firmness as well as a decrease of wrinkle depth and lipid peroxidation [25]. Another study described the conjugation of shinorine with nanoparticles, aiming to improve the UV-blocking activities of nanoparticles themselves. Singh *et al.* were able to produce sunscreen agents, which were photochemically stable products based on extracts rich in shinorine [26].

**Figure 3:**
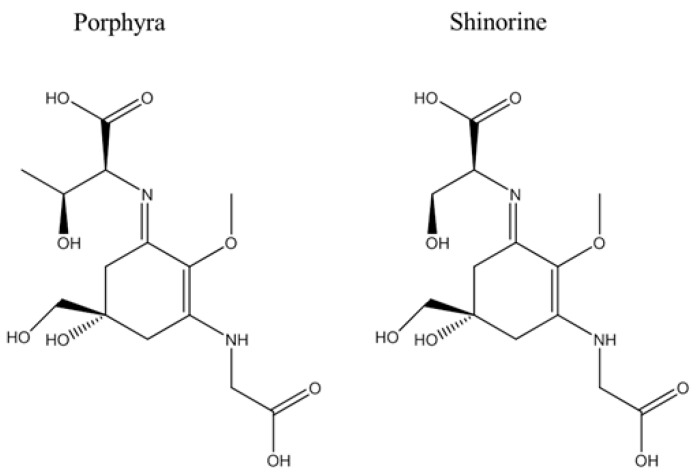
Chemical structures of mycosporine-like amino acids (MAAs), which were isolated from *Porphyra* sp.

Herein, we focus on the bioactivities of two MAAs, porphyra-334 (CID: 101926677) and shinorine (CID: 101926676), which belong to the most abundant MAAs and can be synthetized by numerous algae and cyanobacteria [27,28]. Both structures are based on a cyclohexenimine scaffold with a glycine moiety in position C3, comprising, however, different amino acids in position C1. In the case of shinorine, the structure includes serine, whereas porphyra-334 shows a threonine residue (Figure 3).

Both MAAs were able to modulate inflammatory responses. However, despite structural similarities, there were remarkable differences in the effects.

While a decrease of NF-κB activation would be expected from a compound that is a chemical antioxidant in its strict sense, treatment of cells with shinorine stimulated NF-κB activation significantly and dose-dependently over the whole concentration range. This effect was even more pronounced in unstimulated rather than in LPS stimulated cells. It can be hypothesized that the redox state of the environment influences the activity, whereby an inflammatory environment dampens the stimulatory effect of shinorine.

On the contrary, IDO-1 activity, as indicated by Kyn/Trp was suppressed compared to controls with the lower treatment concentration, but in LPS-stimulated cells only.

Porphyra-334 treatment led to an increased NF-κB activity in unstimulated THP-1-Blue cells over the whole concentration range, although to a lesser extent than shinorine. Interestingly, higher concentrations of porphyra-334 revealed a slight but still significant decrease of NF-κB activation in LPS-stimulated cells compared to LPS control cells. Thus, the activity of porphyra-334 is not only dampened but even reversed in the inflammatory milieu, however, only regarding the activation of NF-κB. Porphyra-334 treatment also affected IDO-1 activity negatively, however, only with the lower treatment concentrations, and for this effect, the stimulation levels of cells seemed not to be of importance.

Despite the structural similarity of both compounds, the mode of action differs in both the NF-κB and the IDO-1 assay. Stability, pH dependency or slight changes in solubility and intracellular localization can contribute to such diverse effects. In fact, de la Coba *et al.* tested different MAAs regarding their antioxidant activity and reported a noticeable influence of the pH on the activity [23]. It could also be hypothesized that the antioxidant activity is not solely the mode of action. Although it is not possible to explain the exact mechanism of action from these experiments, it is of relevance to show that such similar compounds can exert different bioactivities.

Although it would be interesting to decipher the concentration dependent effects of both MAAs in more detail, this study was limited by the availability of pure MAAs for further testing. In addition, changes observed in gene expression were only minor and might be more pronounced after shorter incubation periods.

In future, a more detailed investigation of the NF-κB activation potential of these amino acids would be of interest. NF-κB signaling is firstly discussed as a pro-inflammatory pathway, as it regulates pro-inflammatory cytokine production, leukocyte recruitment, *etc.*, which are important contributors of the inflammatory response. However, the activation of NF-κB signaling in response to stress can also be a strategy of cytoprotection, as several survival pathways can be activated. Anti-apoptotic functions of NF-κB were shown to be protective against inflammation, e.g., in the case of epithelial cell survival and mucosal barrier integrity during persistent leukocyte activation, as demonstrated by Lawrence and colleagues [11]. However, NF-κB can also promote leukocyte apoptosis in certain contexts and contribute to the resolution of inflammation. Thus, NF-κB contributes to the negative feedback control of inflammation, and the spectrum of effects depends on the temporal and spatial regulation of target gene transcription, which is orchestrated by this potent transcription factor. In addition, further recent studies have shown that NF-κB activation is not necessarily pro-inflammatory and also regulates anti-inflammatory pathways [29].

## 4. Materials and Methods

### 4.1. Sample Preparation

**Figure 4:**
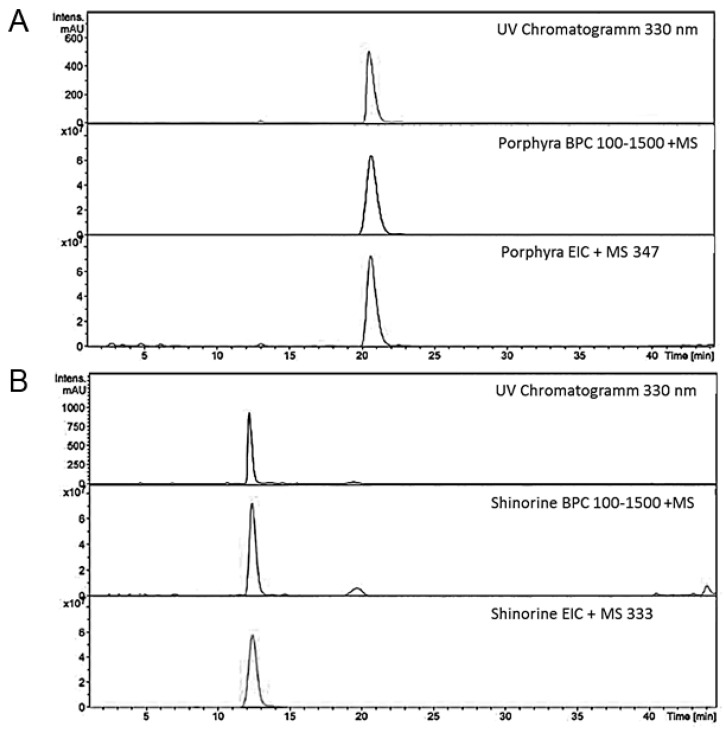
Determination of mycosporine-like amino acid (MAA) purity and identity ((**A**) porpyra-334 and (**B**) shinorine) using liquid chromatography-mass spectrometry (LC-MS); UV-chromatograms were recorded at 330 nm.

For the isolation of the MAAs shinorine and porphyra-334 (Figure 4), the commercially available red alga *Porphyra* sp. containing 10.85 mg/g porphyra-334 and 3.27 mg/g shinorine calculated on dry weight algal material (Asia Express Food, Kilbystraat 1, 8263 CJ Kampen NL) was powdered using a grinding mill prior to extraction with methanol/water (25:75) in an ultrasonic bath (Bandelin Sonorex 35 KHz, Bandelin Electronic GmbH & Co., Berlin, Germany) for 2 h [30,31]. The solution was centrifuged (3000 rpm for 10 min), the supernatant taken aside, and the extraction step repeated two more times; then the pooled solutions were evaporated at 45 °C using a vacuum evaporator (Büchi, Flawil, Switzerland). To obtain completely dried extracts, the resulting pasty liquid was transferred into a beaker and lyophilized (Heto power dry PL 6000, Thermo Fisher Scientific, Waltham, MA, USA).

For purification, the dried extracts were dissolved in water and partitioned three times with 1-butanol before being separated on an ion exchange resin (Dowex 50WXH^+^ form, 100–200 mesh, Sigma Aldrich, Vienna, Austria) according to the protocol of Carignan and colleagues [32]. The MAA fraction was eluted by 0.25 M HCl and then applied on activated carbon cartridges (Supelco Envi-Carb, Sigma Aldrich, Vienna, Austria). They were washed with water, and, finally, the compounds of interest were eluted with pure methanol, receiving a light yellow eluate. Pure MAAs (>95% of purity) were isolated from these pre-purified extracts by semi-preparative HPLC on a Dionex UltiMate 3000 preparative HPLC system (Thermo Scientific, Sunnyvale, CA, USA).

Separation and purification progress was monitored by repeated HPLC-MS analysis (Figure 3), using an HP 1100 HPLC system from Agilent (Waldbronn, Germany) coupled to a Bruker esquire 3000 plus iontrap mass spectrometer (Bruker Daltonics, Bremen, Germany). Therefore, a Luna C-18 column (250 × 3.00 mm, 5 µm, Phenomenex, Torrance, CA, USA) was used with a mobile phase comprising 0.1% acetic acid in water (A) and acetonitrile (B). The method was run isocratically for 30 min with 2% mobile phase B. Then, the column was washed for 10 min with 90% B, before being re-equilibrated for 15 min. The flow was set to 0.5 mL/min. MS-data were obtained in alternating (electrospray ionization (ESI) mode by setting the temperature to 350 °C, the nebulizer gas (nitrogen) to 40 psi, and a nebulizer flow (nitrogen) of 8 L/min. The scanned mass range was between *m/z* 100–1500 at a capillary voltage of 4.5 kV.

### 4.2. Structural Analysis of MAA

In addition to the LC-MS experiments, NMR spectra were recorded at 25 °C on an Ultra-Shield Bruker 600 MHz instrument (Bruker BioSpin, Rheinstetten, Germany) to assure identity and purity of the compounds. The following experiments were performed: ^1^H and ^13^C NMR, two dimensional correlation spectroscopy (2D COSY), heteronuclear multiple quantum coherence (HMQC) and heteronuclear multiple bond coherence (HMBC) spectroscopy. All samples were dissolved in deuterated water D_2_O (99.90%), and tetramethylsilan (both from Euriso-Top, St-Aubin Cédex, France) was used as internal standard. Shift values of the isolated substances were already published in a previous article [6].

### 4.3. Cell Culture

THP-1 human monocytic leukemia cells (DSMZ, Braunschweig, Germany) and the NF-κB/AP-1 reporter cell line THP-1-Blue (Invivogen, San Diego, CA, USA) were cultured in the RPMI 1640 medium completed with l-glutamine (300 mg/L, Biochrom, Braunschweig, Germany) and 10% heat-inactivated fetal bovine serum (FBS, Biochrom, Braunschweig, Germany) at 37 °C in a humidified atmosphere containing 5% CO_2_. THP-1-Blue cell medium contained the selection antibiotic zeocin (Invivogen, San Diego, CA, USA) at a final concentration of 200 µg/mL. For experiments, antibiotic-free medium was used.

### 4.4. Measurement of NF-κB Activation and IDO-1 Activity

THP-1-blue reporter cells contain a construct composed of secreted embryonic alkaline phosphatase (SEAP) under control of a promoter sequence containing NF-κB and AP-1 transcription factor binding sites [33]. Lipopolysaccharide (LPS, Sigma Aldrich, Vienna, Austria) was used to activate NF-κB, which induced SEAP expression. SEAP is secreted in the supernatant and can be quantitatively detected with Quanti-Blue reagent (Invivogen, San Diego, CA, USA). For experiments, cells were plated at a density of 1.6 × 10^6^ cells/mL and pre-treated for 30 min with increasing concentrations of shinorine or porphyra-334 in the range from 12.5–200 µg/mL. Then, cells were stimulated or not with 1 µg/mL LPS. After 48 h, SEAP levels were estimated after incubating 10% (*v/v*) of cell supernatant with 90% (*v/v*) of Quanti-Blue reagent for 120 min, then the signal was measured at a wavelength of 635 nm (Bio-Tek Instruments, Winooski, VT, USA). Concentrations of tryptophan and kynurenine were measured in cell supernatants using high pressure liquid chromatography (HPLC) according to a previously established protocol [34]. The kynurenine to tryptophan ratio (Kyn/Trp), which can be used as an estimate IDO-1 activity, was expressed in μmol kynurenine/mmol tryptophan [16].

### 4.5. Expression of NFKB1 and IL1B

1 × 10^6^ THP-1 cells/mL were treated with 200 µg/mL shinorine or porphyra-334 or were left untreated. After 30 min of incubation, cells were stimulated or not with 1 µg/mL LPS and were incubated for 48 h until being harvested. For RNA isolation, Qiagen RNeasy kit was used according to manufacturer´s instructions (Qiagen, Hilden, Germany).

cDNA was synthesized from 1 µg total RNA using random primers and 20 units Tetro Reverse Transcriptase (Bioline, London, UK). qPCR analysis was performed in a total volume of 15 µL using 15 ng cDNA, 400 nM specific primers for NFKB1 and IL1B and SensiMIX SYBR Lo-ROX reaction kit (Bioline, London, UK) on the Rotor-gene-Q (Qiagen, Hilden, Germany). qPCR reaction was performed under following conditions: 95 °C 10 min; 40 cycles: 95 °C 15 s, 60 °C 15 s (fluorescence acquisition), 72 °C 15 s; melting curve: 95 °C 2 min, 60 °C 5 s, 95 °C 5 s. All cDNAs were analyzed in duplicates. For shinorine, four independent measurements could be performed, however, due to limited availability, for porphyra-334, the experiment could be performed only twice. For each primer pair, the PCR amplicon length was verified once by gel electrophoresis and sequencing. Subsequently, primer specificity was controlled by analysis of melting curves.

The ribosomal protein L37A (RPL37A) was used as an internal reference for normalization in relative quantification, as it has been evaluated as a reliable reference gene in THP-1 cells [35]. The relative expression ratio (R) of the target gene was calculated based on the Ct deviation of the treated sample (TS) from the control sample, normalized to the reference gene (RPL37A) based on the mathematical model proposed by Pfaffl and colleagues [36]. For statistical analysis, the REST software was used (REST ©2009 by Corbett Research Pty Ltd./Qiagen group, Sydney, Australia, and M.W. Pfaffl).

### 4.6. Statistics

Results are expressed as means ± standard error of the mean (SEM). Because some of the data sets did not show normal distribution, group comparisons were performed using non parametric tests (Friedman- and Mann-Whitney U-test). *p*-values < 0.05 were considered to indicate significance.

## 5. Conclusions

Our data indicate the potential of the MAAs shinorine and porphyra-334 to interfere with NF-κB activation, yet further downstream effects are not analyzed. In addition, IDO-1 activity is a potential target. Thus far, recent studies highlight the antioxidant activity and low toxicity of MAAs [8,23]. In our study, both the cellular environment and treatment dose were shown to affect the outcome. Activating inflammatory pathways in an unstimulated context could lead to potential adverse effects due to the promotion of inflammation, while anti-inflammatory properties would dampen immune activation. In conclusion, we recommend a more detailed risk-benefit assessment for these compounds to explore their risks and benefits in combination with their possible therapeutic potential and to reconsider these activities before using MAAs on a larger scale in daily care products.

## Figures and Tables

**Table 1:** Expression levels of nuclear factor kappa nuclear factor kappa B subunit 1 (NFκB1) and interleukin 1B (IL1B) transcripts in lipopolysaccharide (LPS)-stimulated compared to unstimulated THP-1 cells (**A**); in THP-1 cells after treatment with 200 µg/mL of shinorine or porphyra-334 (**B**); and in cells treated with LPS and mycosporine-like amino acids (MAAs) (**C**). Experiments were performed in duplicates in 4 independent experiments for shinorine (* *p* < 0.05) and in 2 independent experiments for porphyra-334 (#).

**(A) LPS-Treated Compared to Control**		
-	NFKB1	IL1B
expression	3.297	92.893
std. error	2.364–5.062	9.594–351.935
*p*-value	0.000 *	0.000 *
-	-	-
**(B) MAA-Treated Compared to Control**		
shinorine		
-	NFKB1	IL1B
expression	1.034	1.193
std. error	0.895–1.222	0.132–7.152
*p*-value	0.568	0.777
porphyra-334 ^#^		
-	NFKB1	IL1B
expression	1.153	1.193
std. error	1.033–1.289	0.916–1.572
*p*-value	0.036 *	0.325
-	-	-
**(C) MAA + LPS-Treated Compared to LPS**		
shinorine (+ LPS)		
-	NFKB1	IL1B
expression	0.823	1.017
std. error	0.599–1.109	0.458–2.479
*p*-value	0.178	0.958
porphyra-334 (+ LPS) ^#^		
-	NFKB1	IL1B
expression	0.851	1.373
std. error	0.650–1.114	0.537–3.605
*p*-value	0.249	0.466

## Abbreviations

The following abbreviations are used in this manuscript:

AP1	activator protein
IDO-1	indoleamine 2,3-dioxygenase
IFN-α	interferon gamma
LPS	lipopolysaccharide
MAA	mycosporine-like amino acid
NF-κB	nuclear factor kappa B
ROS	reactive oxygen species
SEAP	secreted embryonic alkaline phosphatase
